# Tooth loss in middle-aged adults with diabetes and hypertension: Social determinants, health perceptions, oral impact on daily performance (OIDP) and treatment need

**DOI:** 10.4317/medoral.22176

**Published:** 2018-02-25

**Authors:** Fabiana-Barros-Marinho Maia, Emerson-Tavares de Sousa, Fábio-Correia Sampaio, Cláudia-Helena-Soares-de Morais Freitas, Franklin-Delano-Soares Forte

**Affiliations:** 1 Department of Public Health and Clinic Dentistry, Health Sciences Center, Federal University of Paraiba, João Pessoa, PB, Brazil; 2 Department of Pediatric Dentistry, Piracicaba Dental School, Campinas University, Piracicaba –SP/Brazil

## Abstract

**Background:**

This study aimed to explore the association between tooth loss and social determinants, health self-perceptions, OIDP and self-concept of dental treatment need in middle-aged adults with diabetes and hypertension.

**Material and Methods:**

A cross-sectional study was developed with 212 hypertensive and diabetic middle-aged adults (50-65 years). Data were collected from clinical examinations (DMFT) and a questionnaire regarding socioeconomic status, dental health assistance, self-perceptions of oral and general health, OIDP, and the self-concept of dental treatment need. Tooth loss was dichotomized considering the cutoff point of 12 (Model I) or 24 missing teeth (Model II). Data were analyzed using Chi-square, Fisher’s exact test and logistic regression (*p*≤0.05).

**Results:**

Tooth loss was significantly associated with variables such as last dental visit, reason for dental visit, OIDP, perception of dental treatment need, and general self-perception (Model I). Schooling, last dental visit, oral health self-perception and perception of dental treatment need were significantly associated with tooth loss in the Model II. When Model 1 and 2 were adjusted, they demonstrated that last dental visit and perception of dental treatment need were predictor variables.

**Conclusions:**

The annual dental visit and the self-concept of dental treatment need were associated with tooth loss, demonstrating that these variables reduce the tooth loss prevalence.

** Key words:**Access /barriers to care, Dental treatment, Geriatric dentistry.

## Introduction

Increasing longevity is a trend in developed and developing countries around the globe (1). This phenomenon has influenced several aspects that modify the social, political, economic and biomedical dynamic (2). In public health, this multifaceted dynamics considered a challenge to society, must be understood in a holistic way. In this context, the global health scenario has shown an epidemic prevalence of chronic diseases that represents 60% of global deaths, mainly related to hypertension and diabetes (3). Thus, patients with these conditions need to be understood to enable the prevalence and consequences of these illnesses to be monitored, controlled and reduced. This perspective demands multisectoral politics and the investigation of factors capable of influencing the lives of people, such as behavior, social status, personal perception and cultural aspects (4).

Recently, the WHO worked on drafting policies addressing chronic conditions; however, these actions did not consider some aspects of fundamental care, including oral health (5). During the aging process, the cumulative nature of commonly reported oral diseases (dental caries and periodontitis) can promote a significant pattern of severity that culminates in tooth loss. Extensive tooth loss is responsible for physiological and psychosocial complications that are considered a public health problem (6). This issue can be more critical in individuals of disadvantaged countries and those who suffer from chronic conditions.

Oral diseases have a complex aetiopathogenesis that can be associated with the systemic health status (7). Previous studies have shown that oral health status is negatively related to hypertension (8) and diabetes (9-11), although the causality of these chronic diseases and oral health is controversial. Indeed, it is clear that there are common risk factors that link systemic conditions with oral health, such as the intake of sugar, tobacco, alcohol, low educational level, and poverty (5). Preliminary evidence has suggested that tooth loss was associated with socioeconomic status (12,13), ineffective access to dental services (14), and self-perceptions of health (15); however, the scientific literature does not provide any clear information regarding these factors in individuals with non-communicable diseases.

Accordingly, understanding of the contextual associated factors that promote oral and systemic diseases is a better way to plan strategies and promote comprehensive and effective care at community level. Thus, this study aimed to explore the associations between tooth loss severity and social determinants, health self-perceptions, OIDP and self-concept of dental treatment need in middle-aged adults with diabetes and hypertension.

## Material and Methods

The present investigation adhered to the STROBE statement for reporting cross-sectional studies (16).

-Ethical Considerations 

This study was approved by the Brazilian Ethics Committee of Federal University of Paraíba (CAE 35493114.4.0000.5188) in compliance with the ethical principles of the Helsinki Declaration. All volunteers agreed to participate and signed a term of free and informed consent.

-Study Area 

This study was conducted in Santa Rita/ PB/ Brazil, considered an industrialized and medium-sized city (726 Km2) in northeastern of Brazil. The city has a Human Development Index (HDI) of 0.627 and a Gini index of 0.46 (http://www.pnud.org.br).

Heath care in this region is guided by the Brazilian Unified Health System (SUS) policies. Preventive and therapeutic assistance are provided by a network of 42 Family Health Units (FHU) that give access to 83.81% of the population. The oral health care is present in primary attention by the integration of a general dentist for each 32 family health teams. The FHU works systematically according to free demand and the planning of strategies in each age group. There are many programs of health monitoring and quality of life improvement in middle-aged individuals. Therefore, considering the aim of this article, we focused on two programs: the ‘Hiperdia’ (short for Hypertension/Diabetes) Program (17) and Smiling Brazil (18). The first program was specially designed to diagnose, control and treat the non-communicable disease. The second was created to promote oral health care actions.

-Study Design and Sample 

**Figure 1:**
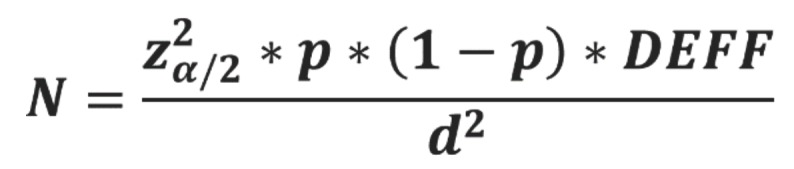
Formula.

A community-based cross-sectional study was conducted, involving a randomly selected sample of 212 hypertensive and diabetic adults, aged between 50 and 65 years. The sample was represented by inhabitants of Santa Rita/PB/Brazil (estimated population: 133.927 inhabitants). The calculation was made according to the following formula, (Fig. 1).

Where α is the probability of type I error, p is the prevalence proportion, DEFF is the estimated effect size and d is the desired level of absolute precision. Thus, sample size estimation assumed that the tooth loss proportion was 0.73 in middle-aged adults with chronic disease (19), an alpha level of 0.05 (two-tailed test), confidence interval of 0.95, Estimated Design Effect (DEFF) of 1, desired level of absolute precision of 0.06, and power of 0.80.

**Figure 2:**
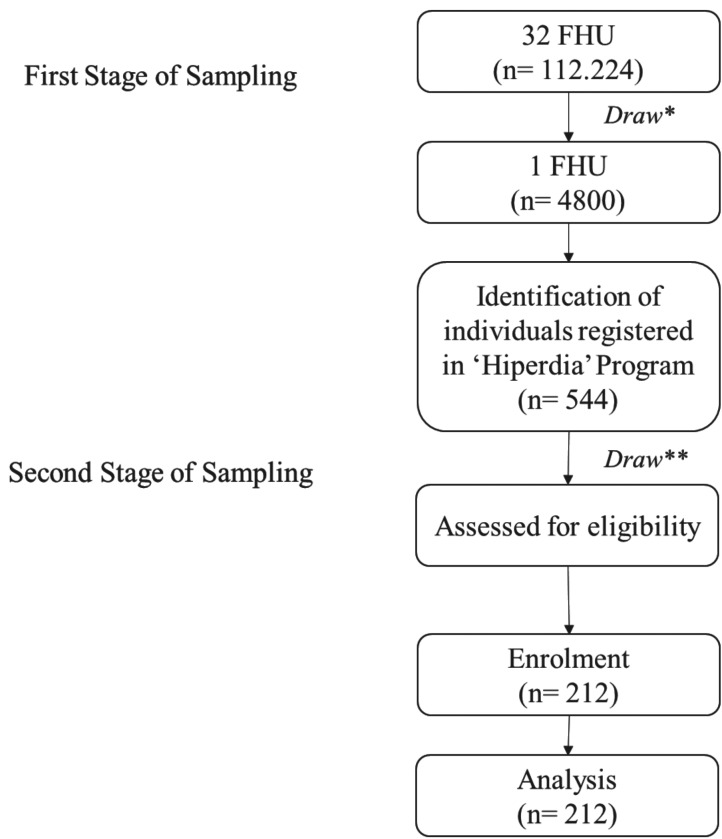
Flow Diagram of sampling procedure and study design. * First Stage of Sampling; ** Second Stage of Sampling.

The double stage conglomerate sampling method was adopted considering the families enrolled in 32 FHU according to information obtained from the Basic Attention Information System (Ministry of Health/SUS). In the first stage, a draw (lottery) among the 32 FHU selected one FHU, composed of 4800 individuals. Among this population, 544 individuals were registered in the ‘Hiperdia’ Program. Thus, the second stage considered the random selection of individuals that would be included according to the eligibility criteria until the desired sample of 212 middle-aged adults was reached (Fig. 2).

The inclusion criteria considered individuals with medical diagnosis of hypertension and diabetes, aged between 50 and 65 years and who signed the consent form. Individuals were diagnosed with hypertension when they had at least two blood pressure measurements above 140/90 mmHg (20). Diabetic individuals were diagnosed when one of the following characteristics were found (21).

- Fasting blood sugar levels above 126 mg / dl with symptoms of polyuria, polydipsia and weight loss.

- Presence of symptoms and the blood sugar levels above 200 mg / dl at any time of the day.

- Absence of symptoms and individuals’ fasting blood sugar levels above 126 mg / dl for more than one measures.

- Individuals with fasting blood sugar values less than 126 mg / dl but with raised levels (greater than 200 mg / dl) 2 hours after the administration of 75 g of oral glucose.

The exclusion criteria considered individuals with physical and/or mental disability, others chronic diseases and those who did not completely fill out the questionnaire or refused to be examined.

-Pilot Study

Initially, the examiner (a dentist) and a dental assistant were trained by an experienced oral epidemiologist (gold standard), in a sample with similar age. The training exercise considered two steps (theoretical and clinical).

• Theoretical Phase: Discussion of DMFT index and study of WHO guidelines for epidemiological oral health surveys (22).

• Clinical Phase: A pilot study conducted with a sample of 30 randomly selected participants who were not included in the main sample. The reliability of dental caries assessment (DMFT) was checked twice within a 2-week period and assessed by the kappa test (k= 0.98). The pilot study results also revealed no misunderstandings regarding the questionnaire methodology and comprehension of it.

-Oral Examinations

Individuals were examined at home, in a face-to-face position, under natural light, using a sterilized mouth mirror, a WHO probe (Golgran Ind. E Com. Ltda., São Paulo, SP, Brazil) and gauze to dry the teeth according to WHO recommendations for epidemiological oral health surveys. The tooth loss variable was defined as any loss of natural teeth due to extraction, for any reason (M component of DMFT index - codes 4 and 5). Tooth loss was considered the dependent variable and was classified into two distinct models.

- Model I: The cutoff point of 12 missing teeth was established, including the anterior arch, based on Batista *et al.* (23). This model was designed considering the shortened dental arch concept, which considers that ten pairs of teeth that occlude, without presenting aesthetic spaces can be considered satisfactory.

- Model II: The dichotomization of tooth loss considering the cutoff point of 24 missing teeth was stated. This model was based on Silva *et al.*, (24) that used the median to dichotomize the severity of tooth loss.

-Questionnaire 

A structured questionnaire was applied to the volunteers at their respective homes. Questions were asked regarding sociodemographic and economic status (age, gender, schooling and household income), dental heath assistance (last dental visit and reason for the last dental visit), general and oral health self-perception (Are you satisfied with your general health? / Are you satisfied with your oral health?), and self-concept of dental treatment need (Do you feel the need for dental treatment?). These covariates were chosen considering the biological plausibility of the contextual aspects of hypertension, diabetes and tooth loss. The categories of each variable were: gender (male and female), schooling (≤ or > 8 years), household income (≤ or > 1 minimum wages – USD 282,161), last dental visit (≤ or > 1 year), reason for the last dental visit (revision or treatment), general health self-perception (great, regular, or poor), dental health self-perception (satisfied, medium, or dissatisfied) and perception of treatment need (yes or no).

-Oral Impact on Daily Performance (OIDP) Questionnaire 

OIDP was evaluated by a validated questionnaire (25). This instrument measured how often during the previous 6 months: the volunteer had problems with their teeth and mouth that caused any difficulty with eating, speaking, cleaning teeth, smiling, sleeping, work performance and social contact. The item “emotional stability” was removed due to problems with translation and possible misinterpretation. Each frequency domain was scored 0-3, where (0) = never, (1) = less than once a month, (2) = once or twice a month up to once or twice a week, (3) = 3-4 times a week or more often. The score of impacts was dichotomized into (1) affected (including the original scores 1, 2, 3 in any domain) and (0) not affected (including the original score 0 for all domains).

-Statistical analysis

Statistical analysis was performed using SPSS software 21.0 (SPSS, Inc., Chicago, IL, USA). The Chi-square (χ2) and Fisher’s exact tests were performed to test the association between variables, as well the odds ratio (OR). The significance level was set at *p* < 0.05 (two-tailed). The ‘enter’ method was used to perform the multivariate logistic regression. The OR and p-value were calculated to perform predictive models. The *p*-values up to 0.20 were established for inclusion in the final model, with adjusted values for the significant covariables. The final level of significance considered *p*≤0.05.

## Results

Among middle-aged adults, in the range age of 50-55, 56-60 and 61-65 represented 25% (n=53), 35% (n=74) and 40% (n=85) of the sample. The rate of response to the questionnaire was 100%. The sample demonstrated a median DMFT of 27.0 (Interquartile Range - IQR: 23.00 – 32.00), with a high number of missing teeth 24.00 (IQR: 16.00 – 32.00). The loss component was experienced in the entire sample, without difference between gender (*p*>0.05).

The binary analysis evidenced that there was significant association between tooth loss and covariables, such as last dental visit, reason for dental visit, OIDP, self-perception of general health and self-concept of dental treatment need (*p*<0.05) – Model I. The Model II demonstrated that schooling, last dental visit, self-perception of oral health, and self-concept of dental treatment need were variables significantly associated with the extensive tooth loss - *p*<0.05 ( Table 1).

**Table 1:**
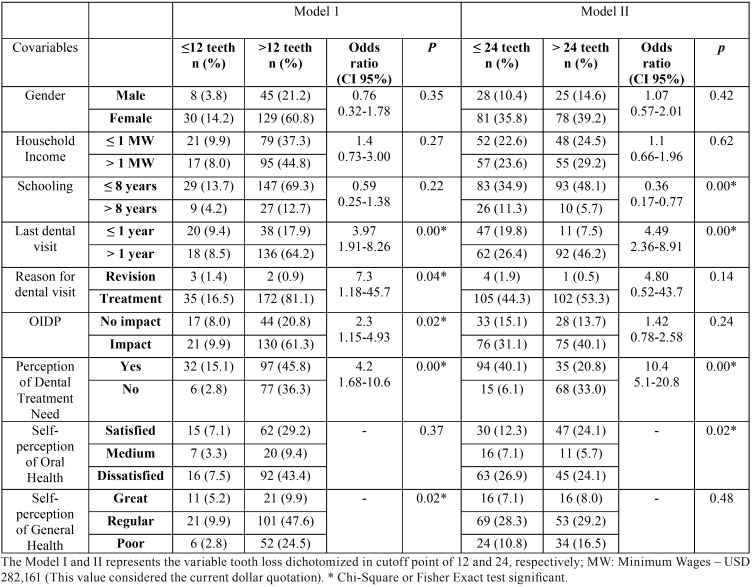
Tooth loss in middle-aged adults with hypertension and diabetes according to two models of severity.

In  Table 2, it was observed that last dental visit, OIDP, and self-concept of dental treatment need were predictable variables (*p*<0.05) in adjusted Model I; and so were schooling, last dental visit and self-concept of dental treatment need in adjusted Model II.

**Table 2:**
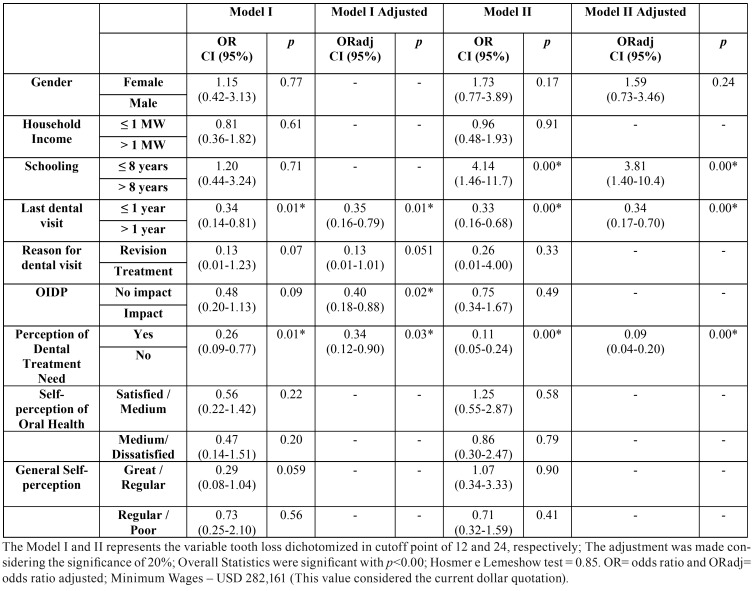
Binary Logistic Regression of tooth loss according to two models of severity.

## Discussion

This research suggested that the chance of tooth loss diminished by 65-66% when the dental visit occurred annually. More importantly, the self-concept of dental treatment need after adjustment for controlling potential confounders was considered a protective factor for tooth loss, reducing the chance of tooth loss by 66 and 91% in Model I and Model II, respectively. This finding was important because it strongly supported the importance of the behavioral pathway of tooth loss in poor (social condition) realities.

This field study showed no association between household income, gender and tooth loss, although schooling was significantly associated with tooth loss in Model II (crude and adjusted). This information emphasizes that the level of instruction can be considered an important route to prevent tooth loss in poor populations that use public health services. Previous studies have demonstrated that socioeconomic variables have a consistent relationship with health status (26,27), tooth loss (6,28) and non-communicable diseases (29). In addition, education can be considered a modifying factor of health outcomes, influencing health knowledge, behaviors, employment, social beliefs, standing, and networks (30,31). Thus, a comprehensive social policy has to integrate progressive and intersectional actions for improvement of pro-poor welfare, thereby reducing social and health inequalities.

There was a high prevalence of tooth loss among the participants, suggesting that a large proportion of the sample may have experienced tooth extraction early in life. Tooth loss prevalence among middle-aged adults with chronic diseases reproduces a reality of poor oral health when compared with a systemically healthy population (12,32). This penchant was also observed in previous researches that demonstrated a significantly higher prevalence of tooth loss in individuals with diabetes (9-11), hypertension (8,33), and metabolic syndrome (19,34). However, it was difficult to estimate how diabetes and hypertension affected oral health due to the several modifying factors. In this perspective, considering that many pathophysiological mechanisms explains the link between diabetes and hypertension (35), it is possible that individuals with these chronic conditions experienced tooth loss more severely than individuals with good systemic health (11). Some factors might be linked with tooth loss, explaining its incidence and severity through different and connected pathways: difficulties of access to health services (14), self-perceptions of oral health (15), the complexity of interactions between oral and systemic diseases (5,36), and social inequality in health (13,37). In this context, the knowledge about the cultural, historical and contextual factors associated with oral care and access to dental services might be a relevant point for research, providing more information relative to the better way to plan interventions in community and individual level. In our study, the main reason for dental consultation was dental extraction in 68.9% of the volunteers. The majority of individuals in our sample only decided to seek a dentist when they felt discomfort, which might be evidence that middle-aged adults did not realize the importance of periodic dental visits. Moreover, the annual dental consultation reduced the chance of tooth loss in individuals with chronic disease. Although the barriers of access to oral health care services were not fully assessed in our investigation, it is suggested that this event occurs due to cost, availability of oral health care services, the length of waiting lists, and fear of dentists.

Dental services have low acceptance among the elderly. This fact is a consequence of individual and community factors such as habitual behaviors, lifestyle, social interaction, oral health policies, health service availability, and socioeconomic structure (38,39). The public health cares in Brazil, accomplished by affirmative policies, are marked by new challenges and persistent difficulties. The reality of our study shows the necessity of expanding the available primary and secondary care, treating these individuals in a holistic way. Moreover, it would be necessary to reinforce the importance attached to general and oral health services, including an annual schedule of evaluation for interdisciplinary team-based care, capable of caring integrally these patients, mainly in vulnerable populations.

This investigation also evaluated the subjective factors associated with tooth loss. This issue is interesting because incorporated the individual perceptions and expectation of treatment into the health problem (15). In this framework, our research demonstrated that some domains such as the self-perception of oral and general health, OIDP and self-concept of dental treatment need were significantly associated with tooth loss in diabetics and hypertensive individuals. However, this data demonstrated a different tendency according to the proposed model. Remarkably, our study showed that tooth loss assessed by Model I showed a significant negative influence on OIDP. On another hand, in Model II (more severe levels of tooth loss) the OIDP did not exhibit any association or predict effect. This outcome reinforces the fact that the responsiveness of OIDP changed according to the severity of tooth loss (40) reflecting that the number of teeth lost was a socio-dental indicator of cultural attitudes and personal understanding of illness (41).

Interestingly, the self-perception of oral and general health did not demonstrate a good predictor variable in the multivariate analysis. On another hand, the self-concept of dental treatment need was associated with less tooth loss, and was also considered a protective and predictor variable for tooth loss. A possible explanation for this finding would be the interactive effects of the sense of coherence supported by their positive association with various aspects of adult oral health (42,43). Thus, considering that the psychosocial impacts of oral disorders tended towards an inter and intra-individual variation, the understanding of the patient’s perceptions is important for the effective planning of health services (44).

Several strengths can be observed in this research, such as the large and specific population-based database with a fair number of independent variables; the high response rate (100%); the use of multivariable analysis that statistically fitted potential confounding factors; and the great internal and external validity, considering the methodological assessment of variables and sampling process. On the other hand, this study had some limitations. Firstly, the study was designed as a cross-sectional survey that cannot provide information regarding temporality. Secondly, the use of questionnaires, naturally, provided a bias’ risk that must be considered, however, to minimize this limitation a pilot study was conducted. Thirdly, we were unable to analyze the outcomes based on the type of diabetes, glycemic status, and the blood pressure. Fourthly, there was absence of a control group (without chronic disease). This limitation made it impossible to identify possible differences between individuals with and without diabetes and hypertension.

## Conclusions

There was a pattern of tooth loss that could be hypothesized according to the annual dental visit and the self-concept of treatment need. These findings had policy implications relative to the planning of health policies considering the social inequalities as part of health strategies in primary care. Moreover, the study reinforced the importance of understanding the individual and community aspects of illness in middle-aged, poor, and chronically affected individuals.
